# Moderating role of positive emotions on emotional reactivity to daily events and negative emotions in older adults

**DOI:** 10.1093/geroni/igaf122.3814

**Published:** 2025-12-31

**Authors:** Rose Lin

**Affiliations:** University of Rochester, Rochester, New York, United States

## Abstract

**Background:**

Daily stressors accumulate to negatively affect emotional well-being and may accelerate cognitive decline in older adults. Emotional reactivity, or heightened emotional responses to stress, is a key contributor. Positive emotions may buffer these effects, yet it remains unclear whether this protective role is preserved across levels of cognitive functioning and decline.

**Objective:**

To test whether positive emotions moderate the association between emotional reactivity to daily events and negative emotions, and whether cognitive ability or cognitive decline influences this effect.

**Methods:**

Participants were 2,041 adults aged 50–94 from the MIDUS study who completed cognitive assessments at two waves (2004–2006, 2013–2014). Emotional reactivity was assessed with the Multidimensional Personality Questionnaire, affect with the Positive and Negative Affect Schedule, and cognition with the Brief Test of Adult Cognition by Telephone. Hierarchical regressions examined the moderating role of positive emotions across cognitive ability and cognitive decline subgroups.

**Results:**

Higher emotional reactivity was significantly associated with greater negative emotions. Positive emotions moderated this association, such that individuals with higher positive emotions showed weaker links between reactivity and negative emotions. This buffering effect was consistent across levels of cognitive ability. However, when stratified by cognitive decline, moderation was observed only among those with high decline, but not moderate decline.

**Conclusions:**

Positive emotions mitigate the impact of stress reactivity on negative mood in later life, even in the presence of cognitive impairment. Findings highlight positive emotion as a resilience factor and support interventions that enhance positive affect to promote emotional well-being.

